# Diet-induced weight loss in obese/diabetic mice normalizes glucose metabolism and promotes functional recovery after stroke

**DOI:** 10.1186/s12933-021-01426-z

**Published:** 2021-12-22

**Authors:** Dimitra Karampatsi, Alexander Zabala, Ulrika Wilhelmsson, Doortje Dekens, Ellen Vercalsteren, Martin Larsson, Thomas Nyström, Milos Pekny, Cesare Patrone, Vladimer Darsalia

**Affiliations:** 1 NeuroCardioMetabol Group, Department of Clinical Science and Education, Södersjukhuset, Internal Medicine, Karolinska Institutet, 118 83 Stockholm, Sweden; 2 Laboratory of Astrocyte Biology and CNS Regeneration, Department of Clinical Neuroscience and Rehabilitation, Center for Brain Repair and Rehabilitation, Institute of Neuroscience and Physiology, Sahlgrenska Academy at the University of Gothenburg, Gothenburg, Sweden

**Keywords:** Type 2 diabetes, Stroke, Insulin resistance, Weight loss, Neurological recovery

## Abstract

**Background:**

Post-stroke functional recovery is severely impaired by type 2 diabetes (T2D). This is an important clinical problem since T2D is one of the most common diseases. Because weight loss-based strategies have been shown to decrease stroke risk in people with T2D, we aimed to investigate whether diet-induced weight loss can also improve post-stroke functional recovery and identify some of the underlying mechanisms.

**Methods:**

T2D/obesity was induced by 6 months of high-fat diet (HFD). Weight loss was achieved by a short- or long-term dietary change, replacing HFD with standard diet for 2 or 4 months, respectively. Stroke was induced by middle cerebral artery occlusion and post-stroke recovery was assessed by sensorimotor tests. Mechanisms involved in neurovascular damage in the post-stroke recovery phase, i.e. neuroinflammation, impaired angiogenesis and cellular atrophy of GABAergic parvalbumin (PV)+ interneurons were assessed by immunohistochemistry/quantitative microscopy.

**Results:**

Both short- and long-term dietary change led to similar weight loss. However, only the latter enhanced functional recovery after stroke. This effect was associated with pre-stroke normalization of fasting glucose and insulin resistance, and with the reduction of T2D-induced cellular atrophy of PV+ interneurons. Moreover, stroke recovery was associated with decreased T2D-induced neuroinflammation and reduced astrocyte reactivity in the contralateral striatum.

**Conclusion:**

The global diabetes epidemic will dramatically increase the number of people in need of post-stroke treatment and care. Our results suggest that diet-induced weight loss leading to pre-stroke normalization of glucose metabolism has great potential to reduce the sequelae of stroke in the diabetic population.

**Supplementary Information:**

The online version contains supplementary material available at 10.1186/s12933-021-01426-z.

## Background

The prevalence of type 2 diabetes (T2D) has been increasing dramatically over the past decades [1] and it is estimated that people with T2D will reach 642 million by 2040 [2]. Since T2D is a strong stroke risk factor [3, 4], current and future diabetic individuals constitute an enormous group in need of potential post-stroke rehabilitative care. T2D is also an established predictor of poor functional outcome after stroke [5, 6], underlining the high medical need in this area.

Over 80% of T2D individuals are overweight or obese and obesity is also a cardiovascular risk factor [7, 8]. Fortunately, body weight reduction in this group reduces cardiovascular risk [9, 10]. Moreover, deliberate weight loss by lifestyle change in unhealthy obese individuals (including those with T2D) who lost at least 10% of their initial body weight, decreases the risk of all-cause mortality by 16% [11] and cardiovascular disease endpoints by 21% [12], likely by reducing cardiometabolic risk factors such as hyperinsulinemia and hyperglycemia [13]. In patients after bariatric surgery, the total mortality is further reduced by 29%, stroke by 34%, and myocardial infarction by 29% [14]. Although weight loss appears to be a potentially effective strategy for stroke prevention in T2D people [15], whether post-stroke rehabilitation can also be improved by this approach remains to be investigated.

Weight loss before stroke could enhance post-stroke functional recovery by improving glycemia. Admission hyperglycemia is associated with impaired recovery in people with and without T2D [16–18] and poor glycemic control in T2D increases stroke mortality [19]. Since weight loss reduces hyperglycemia [20], strategies decreasing the body weight could also improve post-stroke recovery. Peripheral insulin resistance (IR) is a prevalent metabolic complication and a strong predictor of age-related diseases, such as hypertension, stroke and obesity/T2D [21]. Weight loss reduces IR [22, 23] but studies investigating whether this strategy can also improve post-stroke recovery are lacking.

Neuroinflammation, angiogenesis and the modulation of GABAergic activity are cellular mechanisms implicated in stroke recovery that, among others, could also be affected by weight loss interventions. In fact, obesity and T2D are recognised as states of low-grade, chronic systemic inflammation [24, 25] and T2D/obesity-associated overnutrition leads to neuroinflammation [26]. Although the role of neuroinflammation in stroke is very complex, several studies have described the presence of neuroinflammatory responses, both acutely and in the chronic phase after stroke [27]. Moreover, reactive astrocytes, which are associated with neuroinflammation are thought to play multiple roles in the post-stroke functional recovery, e.g. they are important for the protection of the ischemic penumbra [28–30] or inhibit axonal sprouting and possibly also post-stroke synaptic plasticity that allows functional re-mapping [31, 32]. We have recently shown that T2D/obese mice exhibit increased neuroinflammation after stroke compared to non-T2D mice [33]. However, whether pre-stroke weight loss in T2D decreases stroke-induced neuroinflammation remains to be investigated.

The angiogenic response to ischemic brain injury correlates with improved functional outcome after stroke, in both rodents and humans [34]. Moreover, there is emerging evidence that T2D impairs this stroke-induced reparative neovascularization process, impeding functional recovery [35–37]. Whether pre-stroke weight loss can enhance neurological recovery after stroke by regulating angiogenesis remains to be investigated.

The modulation of GABAergic activity plays a central role in facilitating functional recovery after stroke [38] and we recently showed the association between T2D-induced cellular atrophy of GABAergic neurons positive for parvalbumin (PV) with poor functional stroke recovery [37, 39]. Whether weight loss counteracts this effect remains to be determined.

The aim of this study was to determine whether a pre-stroke dietary change resulting in weight loss in T2D/obese mice improves post-stroke functional recovery. We compared the effects of a short- versus a long-term dietary change to determine whether body weight reduction is sufficient to improve functional recovery or whether the complete normalization of glycemia and peripheral IR is necessary. Finally, we determined whether pre-stroke dietary change leading to weight loss inhibits specific mechanisms involved in neurovascular damage aggravated by T2D in the post-acute phase after stroke: neuroinflammation, impaired angiogenesis and atrophy of PV+ interneurons.

## Methods

The work follows the 2010/63/EU directive and has been approved by the regional ethics committee (approval ID1126, Karolinska Institutet). The study is reported according to the ARRIVE guidelines [40].

### Sample size calculation

Group sizes were determined based on 20 ± 10%, anticipated difference between groups in functional recovery with α = 0.05, statistical power of 90%. Standard deviation used in sample size calculation was obtained from pilot experiments. The analyses suggested the sample size of minimum n = 5 per group. However, after taking into consideration the success rate of stroke surgery, mortality and likelihood of statistical outliers, the experimental groups were set at n = 10–15 each.

### Animals

Eighty-one male C57BL/6JRj mice (Janvier Labs, France) were used in this study. Mice were housed in environmentally controlled conditions (22 ± 0.5 °C, 12/12 h light/dark cycle with ad libitum access to food and water). The mice were housed under pathogen free conditions in type III size individually ventilated cages with wood chip bedding and nest material.

### Experimental design

The aim of our study was to compare the effect of a short- and a long-term dietary change leading to weight loss before stroke on post-stroke functional recovery. Although a head-to-head comparison of short- or long-term diet control would have been desirable, the age difference between mice after short- or long-term diet control would not have allowed a fair comparison. Therefore, we performed two independent studies with either a short- or long-term diet intervention groups having their own age-matched control groups exposed to high fat diet (HFD) or standard diet (SD) for the same time duration.

#### Study 1 (short-term dietary change)

**Fig. 1:**
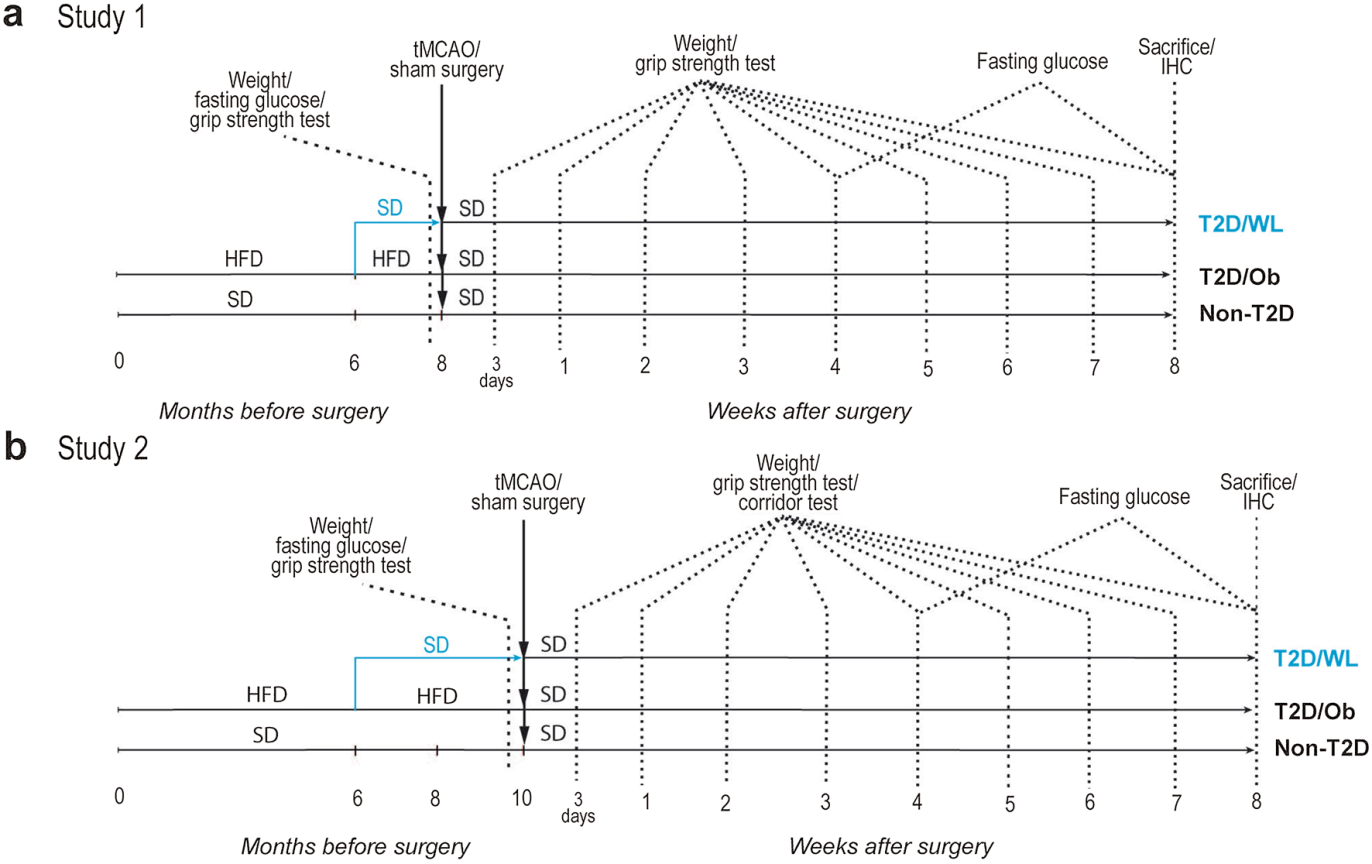
Experimental design. **a** Experimental design of Study 1 (short-term dietary change). **b** Experimental design of Study 2 (long-term dietary change)

Thirty-six mice were used. From 4 weeks of age, the mice were fed with either SD (*n* = 10, hereafter referred to as non-T2D group) or HFD (60% energy from fat) (*n* = 26, hereafter referred to as T2D/Ob group) for 6 months to induce obesity (over 25% BW increase) and T2D features (fasting glucose over 7 mmol/L and insulin resistance). After 6 months of exposure to HFD, to induce weight loss in a sub-group of T2D mice, HFD was replaced with SD (n = 15, hereafter referred to T2D/WL group), while the rest of T2D mice remained on HFD (n = 11). After the weight loss period of 2 months, fasting glucose, insulin sensitivity and body weight were measured and then the mice were subjected to transient middle cerebral artery occlusion (tMCAO) (see below). After tMCAO, the HFD was also substituted with SD in the T2D group, to reflect the clinical setting of a balanced post-stroke diet. See Fig. 1 for the experimental design.

One mouse from the non-T2D group was removed due to unsuccessful tMCAO. Two mice were removed from the T2D group (euthanised) shortly after surgery, since their condition reached a humane endpoint as specified in the ethical approval and two mice were removed from WL group due to unsuccessful tMCAO. The final number of animals used in this study were as following: non-T2D (n = 9), T2D/Ob (n = 9) and T2D/WL (n = 13). The forelimb sensorimotor function was then measured by assessing weekly upper-limb grip strength (see below) for 8 weeks after stroke when the mice were sacrificed (time when the non-T2D mice have fully recovered). Brains were then collected for histology. See Fig. 1 for the experimental design.

#### Study 2 (long-term dietary change)

Forty-five mice were used. From 4 weeks of age, the mice were fed with either SD (*n* = 15) or HFD (*n* = 30) for 6 months. After 6 months of exposure to HFD, in a sub-group of T2D mice, HFD was replaced with SD (n = 15), while the rest of T2D mice remained on HFD (n = 15). After a weight loss period of 4 months, fasting glucose, insulin sensitivity and body weight were measured and then mice were subjected to tMCAO or sham surgery (see below). After tMCAO, the HFD was also substituted with SD in the T2D group, to reflect the clinical setting of a balanced poststroke diet. See Fig. 1 for the experimental design.

Eleven mice were removed due to either unsuccessful tMCAO or after reaching a humane endpoint. The final number of animals used in this study were as following: Sham non-T2D (n = 5), sham T2D/Ob (n = 5), sham T2D/WL (n = 5), stroke non-T2D (n = 7), stroke T2D/Ob (n = 6) and stroke T2D/WL (n = 6). The forelimb sensorimotor function was then measured by assessing weekly upper-limb grip strength for 8 weeks after stroke. We also assessed lateralized sensorimotor integration by the corridor text (see below). The mice were then sacrificed and the brains were collected for histology. See Fig. 1 for the experimental design.

### Transient middle cerebral artery occlusion

Stroke was induced by tMCAO by the intraluminal filament technique as previously described [41]. Briefly, mice were anesthetized by inhalation of 3% isoflurane. Anesthesia was maintained by 1.5% isoflurane throughout the surgery. Body temperature was maintained at 37–38 °C using a heated pad with feedback from rectal thermometer. Left external (ECA) and internal (ICA) carotid arteries were exposed and a 7–0 silicone-coated monofilament (total diameter 0.17–0.18 mm) was inserted into the ICA until it blocked the origin of the MCA. Then the wounds were temporarily closed, and the mice were allowed to wake up. After 30 min, the occluding filament was removed. Stroke induction was considered unsuccessful when the occluding filament could not be advanced within the internal carotid artery beyond 7–8 mm from the carotid bifurcation or if mice lacked symptoms of neurological impairment based on the neurological severity score [42]. All mice were given analgesic (Carprofen, 5 mg/kg) and soft food after the surgery.

### Fasting glycemia and insulin tolerance test (ITT)

Fasting glycemia was measured using blood from tail tip puncture, after an overnight fasting. For ITT, mice were fasted for 6 h and injected intraperitoneally with human insulin (0.5 unit/kg) in saline. Blood glucose was measured before insulin injection and at 15, 30, 45, 60, 75 and 90 min thereafter. For analyses, the area under the curve (AUC) was computed for each mouse.

### Assessment of the sensorimotor function

#### Forelimb grip strength

The forelimb grip strength [39, 43] was measured by using a grip strength meter (Harvard apparatus, MA, USA) before, at 3 days and at 1–8 weeks after tMCAO. Briefly, mice were firmly held by the body and allowed to grasp the grid with the right forepaw. Mice were gently dragged backward until the grip was broken. Ten trials were performed, and the highest value was recorded as described previously [39, 43].

#### The corridor task

The corridor task was performed in a 50 cm long, 4 cm wide, and 15 cm high Plexiglas corridor to assess the lateralized sensorimotor integration [44]. The mice were first habituated to the corridor for 5 min for 2 days before the test. On the day of testing, mice were fasted for 6 h. Mice were first habituated for 5 min in an empty corridor and then immediately transferred to an identical corridor containing 10 pots on each side, each containing a flavoured treat. The number of explorations made by the mouse at the ipsilateral or contralateral side was counted for 5 min. The data are presented as the ratio of ipsilateral/contralateral explorations.

The behavioural tests were performed by an experimenter blinded to the experimental groups, although during the first few weeks after tMCAO, group identities were apparent due to the obvious weight differences.

### Immunohistochemistry

The mice were terminally anesthetized with an overdose of sodium pentobarbital and transcardially perfused with saline followed by 4% ice-cold paraformaldehyde. Brains were removed and after overnight post-fixation transferred to a solution of phosphate-buffered saline (PBS) with 20% sucrose until they sank. The brains were then cut in 30-μm-thick coronal sections using a sliding microtome and stored in anti-freeze solution at − 20 °C.

Tissue staining was performed using the free-floating method. Briefly, brain sections were washed in PBS. For staining with 3,3′-Diaminobenzidine (DAB) visualisation, sections were incubated with PBS containing 3% H_2_O_2_ and 10% methanol for 20 min at RT to quench endogenous peroxidases. For both DAB and immunofluorescence stainings, sections were then blocked in PBS containing 3–5% appropriate normal serum and 0.25% Triton-X100 for 1 h (at RT), and incubated overnight in primary antibody solution (at RT for the CD31 staining and at 4 °C for all other stainings).

After overnight incubation with the primary antibody solution, sections were washed and incubated for 2 h at RT with the secondary antibody. For DAB staining, incubation with biotinylated secondary antibody was followed by incubation with avidin–biotin complex (1:200 dilution for both reagent A and B, Vectastain Elite ABC kit, Vector Laboratories) for 1 h at RT, and development with DAB. For more details about primary and secondary antibodies used, see Additional file 1.

### Quantitative microscopy and image analysis

#### Ischemic volume measurement

Ischemic volume was measured using all serial sections with visible stroke. Briefly, NeuN-labelled brain sections with stroke damage were displayed live on a computer monitor. The volumes of the whole contralateral uninjured hemisphere as well and the volume of the intact region of the ipsilateral hemisphere were measured, using Cavalieri estimator probe (MBF Bioscience, USA). To compensate the stroke-induced tissue shrinkage, ischemic volume was calculated by subtracting the volume of intact region of ipsilateral hemisphere from the volume of the whole contralateral hemisphere.

#### Assessment of neuroinflammation

Image analyses using Adobe Photoshop 2021 software were used to evaluate Iba-1 and CD68 immunoreactivity. Briefly, images of Iba-1 staining were acquired at 4× magnification using the Olympus BX51 microscope. Striatum was isolated on images and converted into gray-scale (8-bit) mode and thresholded. Lowest Iba-1 immunoreactivity in sham mouse from each group was used as baseline. After the baseline was set, this preset was used to detect Iba-1 immunoreactivity in contralateral and ipsilateral to stroke striatum, and pixel density was calculated. Values were then normalized based on the respective sham group and expressed as -fold increase over sham. Similarly, CD68 staining was detected on images using Adobe Photoshop 2021 software and quantified in ipsilateral striatum.

A Zeiss Axio Observer wide-field microscope was used to acquire tiled images of GFAP immunoreactivity within striatum using 10× objective. Striatum was delineated in the images using Metamorph image analysis software (Molecular Devices, v. 2.8.5) and thresholded for background subtraction. Integrated density of the thresholded pixels was assessed for striatum of sham and stroked mice and the integrated pixel density for contralateral and ipsilateral striatum post-stroke was normalized to the respective sham group and expressed as -fold increase over sham.

#### Analysis of PV+ interneuron cell body volume

The average soma volume of PV+ interneurons was assessed using the Olympus BX51 microscope connected to the computerized stereology setup from Stereo Investigator (MBF Bioscience, USA). See Additional file 1 for details about this assessment.

#### Analysis of vessel density

For analysis of CD31+ vessels, the drawing tool in Stereo Investigator (MBF Bioscience, USA) was used to delineate the infarct region in the live image (determined based on NeuN staining), after which images of the CD31 staining were acquired at 10× magnification using the Olympus BX51 microscope (two images per striatum). See Additional file 1 for more details about the image analysis.

### Multiplex immunoassay

The levels of plasminogen activator inhibitor-1 (PAI-1) were quantified in mouse serum samples using a mouse Diabetes multiplex magnetic bead kit (Bio-Rad, cat# 171F7001M) and used according to the manufacturer's recommendations (1:4 dilution; 12.5 μL per mouse serum sample). Plates were read using Bio-Plex® MAGPIX™ Multiplex Reader (Luminex) and the results were analyzed using Bio-Plex Manager 6.1 software (Bio-Rad).

### Data and statistical analysis

All data were analyzed by GraphPad Prism Version 9.0. The data were first checked for statistical outliers by using the ROUT method, and normality by using the Shapiro–Wilk normality test to decide whether to perform parametric or non-parametric tests.

*Parametric tests:* Two-way repeated measures ANOVA with Geisser–Greenhouse’s correction followed by Dunnett T3 for metabolic and behavioural tests. One-way ANOVA with Tukey’s post-hoc test was used to analyse the pre-stroke metabolic tests, stroke volume, and parenchymal CD13 density in the ipsilateral striatum. For Iba-1 and CD68 analysis, Brown–Forsythe and Welch ANOVA followed by two-stage linear step-up procedure of Benjamini, Krieger and Yekutieli was used. The same ANOVA test followed by Dunnett’s post hoc test was used for GFAP data analysis. All data are expressed as mean ± SD. A *P*-value less than 0.05 was considered statistically significant.

## Results

### Short- (2 months) and long-term (4 months) dietary change leading to weight loss improves or completely normalizes, respectively, the metabolic profile of T2D mice

**Fig. 2:**
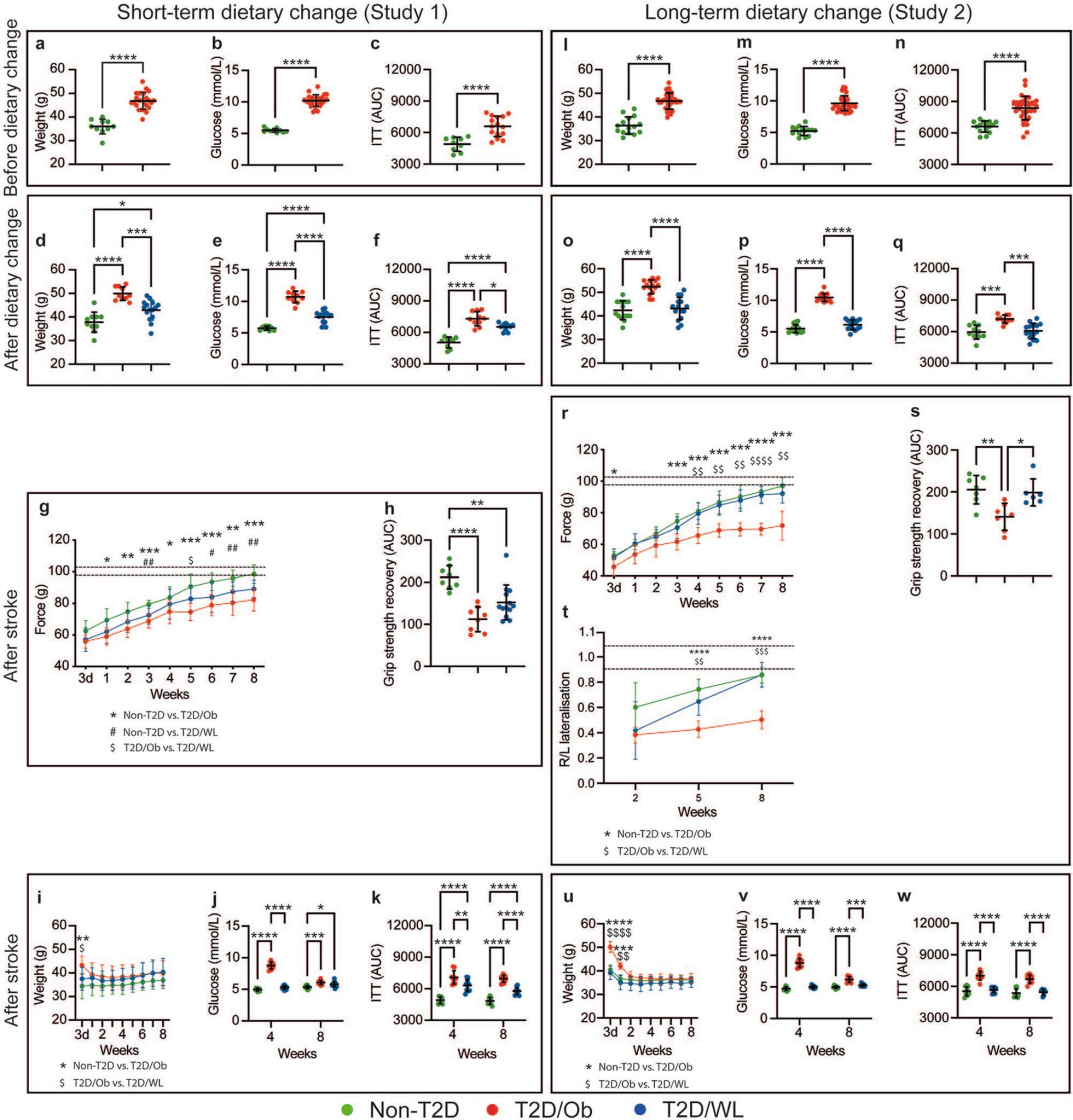
Effects of dietary change on weight, metabolic parameters and functional recovery after tMCAO. **a**–**c** Effect of HFD feeding on body weight (**a**), fasting glucose (**b**) and insulin sensitivity (**c**), Study 1. **l**–**n** Effect of HFD feeding on body weight (**l**), fasting glucose (**m**) and insulin sensitivity (**n**), Study 2. **d**–**f** Effect of short-term dietary change on body weight (**d**), fasting glucose (**e**) and insulin sensitivity (**f**), Study 1. **o**–**q** Effect of long-term dietary change on body weight (**o**), fasting glucose (**p**) and insulin sensitivity (**q**), Study 2. **g**, **h** Forepaw grip strength after stroke shown as a plotted curve (**g**) and AUC (**h**), Study 1. **r**, **s** Forepaw grip strength after stroke shown as a plotted curve (**r**) and AUC (**s**), **t** Corridor task, Study 2. Dashed lines on g, r and t indicate the range of pre-stroke grip strength (**g**, **r**) and Corridor task (**t**). **i**–**k** Body weight (**i**), fasting glucose (**j**) and insulin sensitivity (**k**) after tMCAO, Study 1. **u**–**w** Body weight (**u**), fasting glucose (**v**) and insulin sensitivity (**w**) after tMCAO, Study 2. Data are presented as mean ± SD. **a**–**c**, **l**–**n** Welch’s t-test. **d**–**f**, **o**–**p**, **h**, **s** Brown–Forsythe and Welch ANOVA followed by Dunnett’s T3 multiple comparisons test. **g**, **r**, **t**, **i**, **u** Two-way repeated measures ANOVA followed by Tukey’s multiple comparisons test. **j**, **k**, **v**, **w** Two-way ANOVA followed by Tukey’s multiple comparisons test. */$/# denotes p < 0.05, **/$$/## denotes p < 0.01, ***/$$$ denotes p < 0.001 and ****/$$$$ denotes p < 0.0001. Group sizes: **a**, **b** non-T2D n = 10, T2D/Ob n = 25. **c** Non-T2D n = 10, T2D/Ob n = 15. **d**, **e** Non-T2D n = 10, T2D/Ob n = 10, T2D/WL n = 15. **f** Non-T2D n = 9, T2D/Ob n = 10, T2D/WL n = 10. **g**–**k** Non-T2D n = 9, T2D/Ob n = 9, T2D/WL n = 13. **l**–**n** Non-T2D n = 14, T2D/Ob n = 33. **o**–**q** Non-T2D n = 10, T2D/Ob n = 10, T2D/WL n = 15. **r**–**w** Non-T2D n = 7, T2D/Ob n = 6, T2D/WL n = 6

Six months of exposure to HFD induced obesity, hyperglycemia and IR in both Study 1 (Fig. 2a–c) and Study 2 (Fig. 2l–n). To induce weight loss in a group of mice in both studies the HFD was replaced with SD, which was continued for 2 (Study 1, T2D/WL) or 4 (Study 2, TD2/WL) months.

After the replacement of HFD with SD for 2 months, the T2D/WL group lost significant weight (p < 0.001), reduced fasting glycemia (p < 0.0001) and improved insulin sensitivity (p < 0.05) compared to the mice that were kept on HFD (Fig. 1d–f, T2D/Ob mice). However, all three metabolic parameters (weight, glycemia and IR) remained significantly higher (p < 0.05, p < 0.0001 and p < 0.05 respectively) than those of age-matched healthy, non-T2D controls (Fig. 1d–f). In contrast, the replacement of HFD with SD for 4 months resulted in the complete normalization (same values as age-matched non-T2D controls) of weight, glycemia and IR, so that T2D/WL mice were no longer different from non-T2D controls (Fig. 2o–q).

### Long-term dietary change leading to weight loss before stroke improves post-stroke functional recovery in association with the full normalization of glucose metabolism

After 2- and 4-months of dietary change leading to weight loss, mice were subjected to stroke and functional recovery was tracked over 8 weeks. As reported previously [33, 37, 43], T2D significantly impaired forepaw grip strength recovery in comparison with age-matched non-T2D mice (p < 0.0001 and p < 0.01 in Study 1 and 2 respectively; Fig. 2g, h, r, s). The short-term (2 months) dietary change did not improve functional recovery after stroke. Specifically, the forepaw grip strength recovery of T2D/WL mice remained similar to T2D/Ob mice and significantly lower than age-matched non-T2D (p < 0.01; Fig. 2g, h). On the contrary, the long-term (4 months) dietary change (where the improvement of glucose metabolism was complete, see above), entirely normalized functional recovery after stroke. In fact, the T2D/WL mice showed a similar forepaw grip strength recovery as age-matched non-T2D controls, and a significant improvement (p < 0.05) compared to T2D/Ob mice (Fig. 2r, s). Significant improvement of stroke recovery in T2D/WL mice was also recorded after long-term (4 months) dietary change by using the corridor task at 5 (p < 0.01) and 8 (p < 0.001) weeks post-stroke (Fig. 2t). No differences in stroke-induced brain damage were observed between the groups in both studies (Additional file 1: Fig. S1).

We also compared body weight, fasting glucose and IR between dietary change groups [short- (2 months) vs. long-term (4 months)]. The body weight was similar (Additional file 1: Fig. S2a), but the long-term dietary change reduced hyperglycemia below diabetic threshold of 7 mmol/L (p < 0.001, Additional file 1: Fig. S2b) and reduced IR compared to short-term dietary change (p < 0.05, Additional file 1: Fig. S2c).

In summary, these results indicate that both a short- (2 months) and a long-term (4 months) dietary change before stroke leads to similar weight loss. However, the full pre-stroke normalisation of the metabolic profile (hyperglycemia and IR) induced by long-term but not by short-term dietary change loss seems to be key to improve functional recovery after stroke.

### Persistent IR prevents functional recovery after stroke

To understand which metabolic parameter played a more pronounced role in improving functional recovery post-stroke after long-term dietary change leading to weight loss, we continued to monitor the metabolic profile also after stroke. Since the long-term (4 months) dietary change normalized all metabolic parameters (weight, fasting glucose and IR) already before stroke (Fig. 2o–q), we relied greatly on the short-term (2 months) dietary change paradigm (Study 1) to infer the answer to this question.

In T2D/Ob mice (in both Study 1 and 2), stroke led to a complete normalization of the weight (equal to non-T2D) already within 1–2 weeks after stroke (Fig. 2i, u). In the T2D/WL group after short-term (2 months) dietary change, the stroke also led to the normalization of weight that reached the same values as non-T2D controls already within the first week after tMCAO (Fig. 2i).

In T2D/Ob mice (in both Study 1 and 2), the fasting glucose remained significantly elevated at 4 weeks after stroke and reached normal values (< 7 mmol/L) only by 8 weeks post-stroke (Fig. 2j, v). In the T2D/WL group after short-term (2 months) dietary change, glycemia normalized quickly and reached normal values already at 4 weeks post-stroke (Fig. 2j), similar to the T2D/WL group after long-term (4 months) dietary change (Fig. 2v). Since both T2D/Ob and T2D/WL groups after 2 months of dietary change showed impaired recovery after stroke (Fig. 2g, h) regardless of the fast normalization of weight and of glycemia regulation, it is likely that neither post-stroke weight loss nor post-stroke glycemia normalization can be linked to stroke recovery.

We also monitored IR at 4 and 8 weeks after stroke. Unlike weight and glycemia, the post stroke change of IR after short- (2 months) and long-term (4 months) dietary change was clearly different. Specifically, while after 4 months of dietary change insulin sensitivity was already normal before stroke and remained so also for the whole recovery phase (Fig. 2w), after short-term (2 months) dietary change, T2D/WL mice showed a reduction of IR compared to T2D/Ob mice (p < 0.01 and p < 0.001 at 4 and 8 weeks after stroke respectively; Fig. 2k). However, IR remained significantly higher than age-matched non-T2D controls during the whole duration of the recovery phase (p < 0.0001 at both 4 and 8 weeks; Fig. 2k).

Taken together, these results suggest that IR before stroke and likely also during the recovery phase is a key factor behind impaired recovery in T2D/Ob mice and T2D/WL after a short-term (2 months) dietary change. These results also suggest the association between the reduction of IR after long-term (4 months) dietary change and the improvement of functional recovery after stroke.

### The pre-stroke plasma levels of PAI-1 are significantly downregulated by dietary change leading to weight loss

In order to determine the potential association between additional metabolic markers and stroke recovery induced by dietary change leading to weight loss, we quantified the pre- and post-stroke serum levels of the plasminogen activator inhibitor 1 (PAI-1), which has been associated with the progression of the metabolic syndrome and diabetes, but also with stroke pathophysiology [45]. T2D/Ob mice showed significantly increased pre-stroke levels of PAI-1 (p < 0.0001) vs. non-T2D mice (Additional file 1: Fig. S3a). Remarkably, the dietary change leading to weight loss entirely counteracted this T2D-induced effect (Additional file 1: Fig. S3a). At 8 weeks post-tMCAO, no significant differences between the groups could be observed (Additional file 1: Fig. S3b).

### Pre-stroke dietary change leading to weight loss dampens post-stroke neuroinflammation in T2D mice

**Fig. 3:**
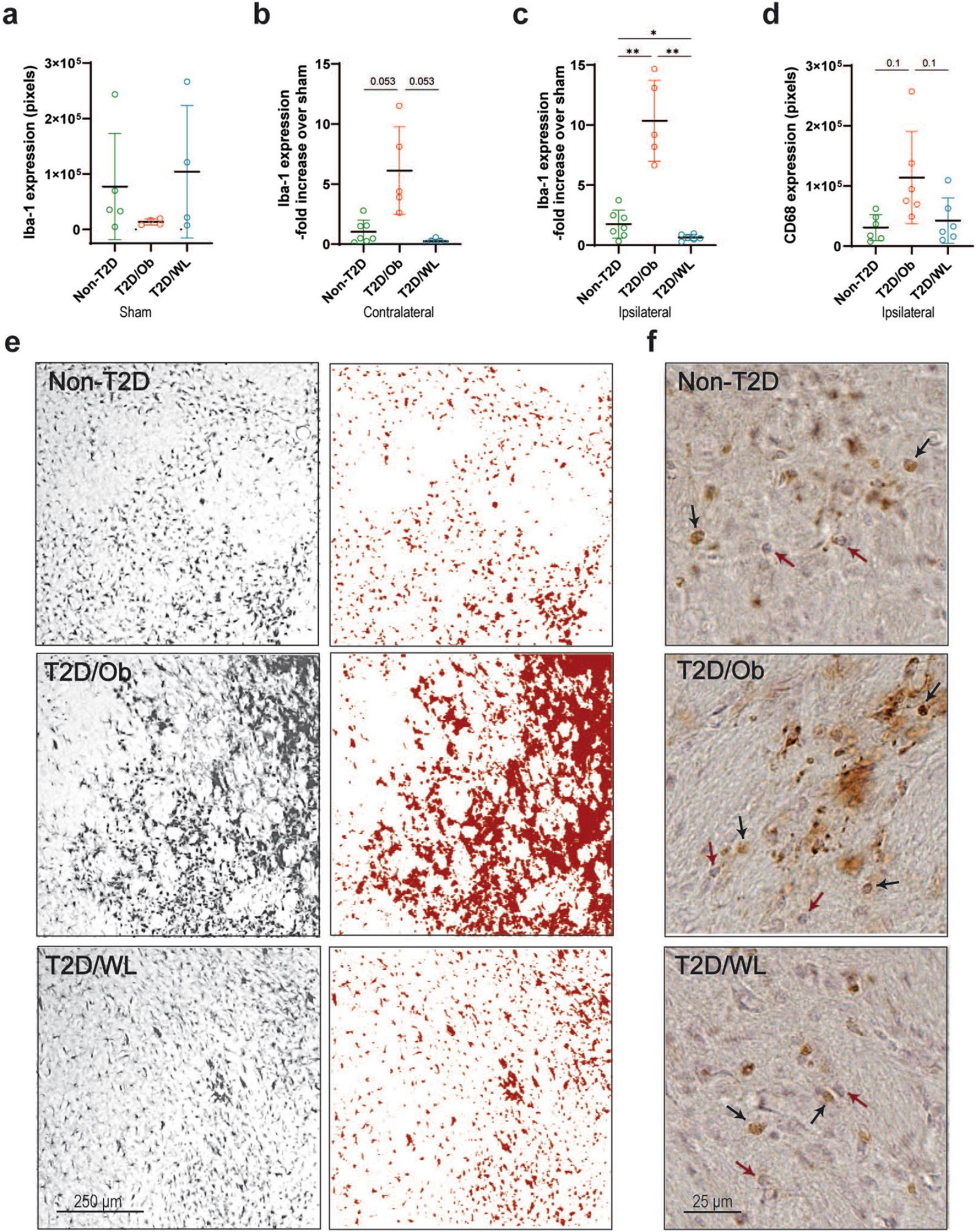
Dietary change leading to weight loss normalizes T2D-induced neuroinflammatory changes after tMCAO. **a** Iba-1 expression in striatum after sham surgery. **b** Iba-1 expression in contralateral striatum after tMCAO surgery. **c** Iba-1 expression in ipsilateral striatum after tMCAO surgery. **d** CD68 expression in ipsilateral striatum after tMCAO surgery. Data are presented as mean ± SD. Brown–Forsythe and Welch ANOVA followed by two-stage linear step-up procedure of Benjamini, Krieger and Yekutieli was used for all analyses. * Denotes p < 0.05, ** denotes p < 0.01. **e** Representative images of Iba-1 staining in ipsilateral striatum after tMCAO (left panel), with corresponding examples of thresholded images (right panel). Scale bar: 250 µm. **f** Representative images of CD68 staining in ipsilateral striatum after tMCAO. Black arrows indicate CD68+ cells and red arrows indicate hematoxylin+ cells. Scale bar: 25 µm. Group sizes: **a** non-T2D n = 5, T2D/Ob n = 4, T2D/WL n = 4. **b**, **c** Non-T2D n = 7, T2D/Ob n = 5, T2D/WL n = 6. **d** Non-T2D n = 6, T2D/Ob n = 6, T2D/WL n = 6

To determine whether the improved functional recovery after stroke induced by long-term (4 months) dietary change leading to weight loss affected stroke-induced inflammation, we assessed the expression of microglia/macrophage markers Iba-1 and CD68 and astrocyte marker GFAP, respectively, in the striatum 8 weeks after stroke. No significant differences were detected at baseline Iba-1 immunoreactivity in sham animals, although a trend towards reduction was observed in T2D/Ob mice (Fig. 3a). To isolate the effect of T2D and dietary change on specifically stroke-induced inflammation, the data from contralateral and ipsilateral striatum of stroke-subjected mice were normalized on the respective shams. As expected, at 8 weeks post-tMCAO, the expression of Iba-1 was still significantly upregulated in ipsilateral vs. contralateral striatum in all groups (data not shown). However, Iba-1 expression was ≈ fivefold greater in the ipsilateral striatum of T2D/Ob mice compared to non-T2D mice (p < 0.01, Fig. 3c, e). Remarkably, weight loss in T2D/WL mice completely abolished this T2D-induced effect (p < 0.01, Fig. 3c, e). A similar pattern as for the ipsilateral striatum was also found in the contralateral striatum although the effect did not reach statistical significance (p = 0.053, Fig. 3b).

Microglia/macrophage activation was then further analyzed by quantifying CD68 expression in ipsilateral striatum. CD68+ cells were not detected in contralateral striatum of any of the groups. However, CD68 expression showed a strong trend toward an increase in ipsilateral striatum of T2D/Ob mice vs. non-T2D mice (p ≈ 0.1, Fig. 3d, f) and this trend was abolished by weight loss in T2D/WL mice (p ≈ 0.1, Fig. 3d, f).

**Fig. 4:**
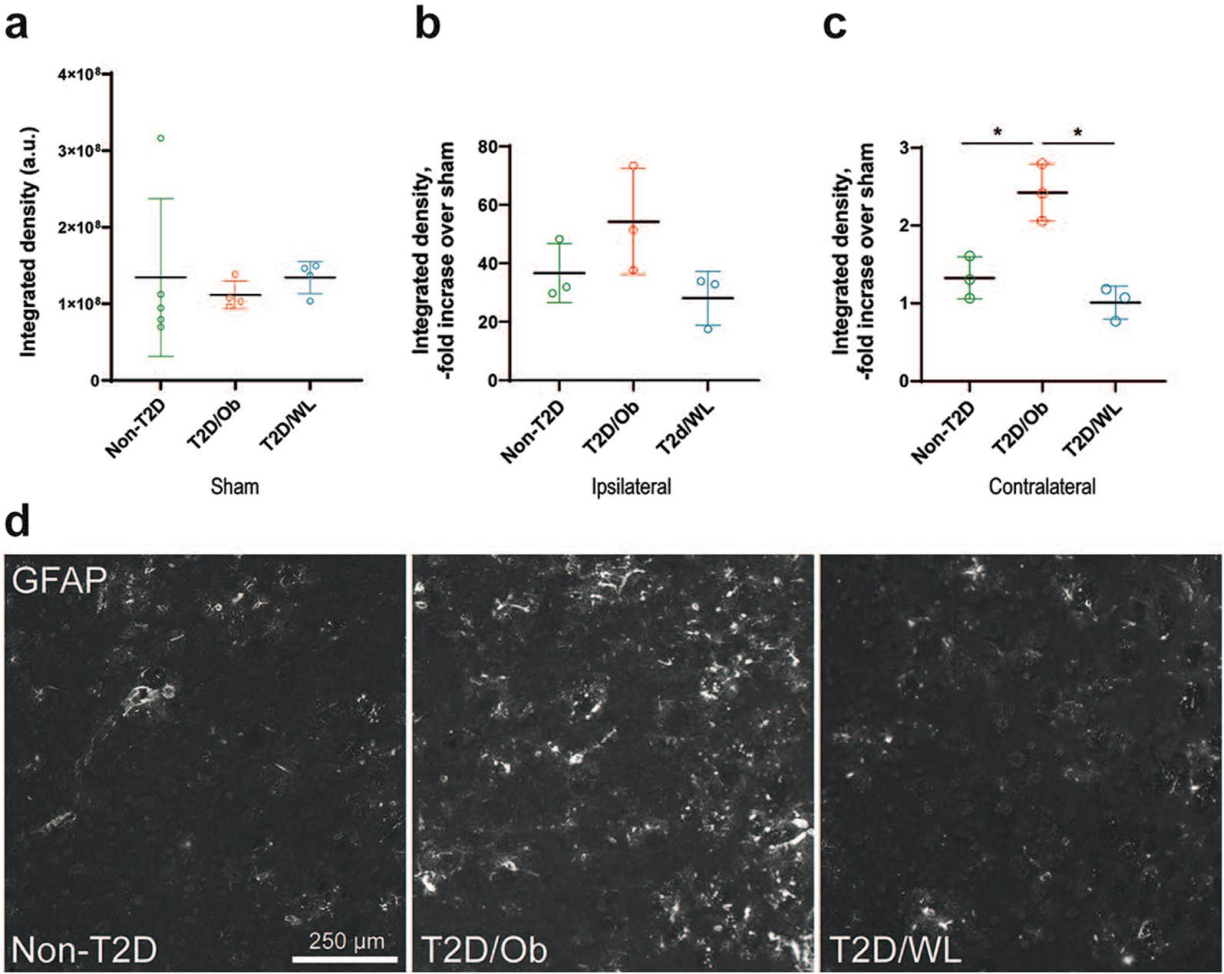
Stroke-induced astrocyte response in contralateral striatum of T2D mice is normalized by weight loss. **a** GFAP immunoreactivity in striatum after sham surgery. **b** GFAP immunoreactivity fold increase over sham in ipsilateral striatum after tMCAO. **c** GFAP immunoreactivity fold increase over sham in striatum contralateral to stroke lesion after tMCAO. Data are presented as mean ± SD. Brown–Forsythe and Welch ANOVA followed by Dunnett’s post hoc test; *p < 0.05. **d** Representative images of GFAP immunoreactivity in contralateral striatum after tMCAO. Group sizes: **a** non-T2D n = 5, T2D/Ob n = 4, T2D/WL n = 4. **b**, **c** Non-T2D n = 3, T2D/Ob n = 3, T2D/WL n = 3

In unchallenged striatum, the GFAP immunoreactivity in astrocytes is sparse, and we detected no difference between the sham groups (Fig. 4a). The GFAP immunoreactivity, normalized to the respective sham group, was highly increased in the infarcted ipsilateral striatum 8 weeks after stroke and was comparable between the dietary groups (Fig. 4b). Most interestingly, the GFAP immunoreactivity in the striatum contralateral to the infarct was increased in the T2D/Ob mice compared to non-T2D (p < 0.05, Fig. 4c, d), indicating signs of neuroinflammation in the contralateral hemisphere. GFAP immunoreactivity in the T2D/WL mice did not differ from the sham group level. These results suggest that improved functional recovery after stroke after long-term (4 months) dietary change leading to weight loss is associated with a decreased neuroinflammatory response with respect to both microglia activation and astrocyte reactivity.

### Pre-stroke dietary change leading to weight loss counteracts the T2D-induced atrophy of parvalbumin-positive interneurons

**Fig. 5:**
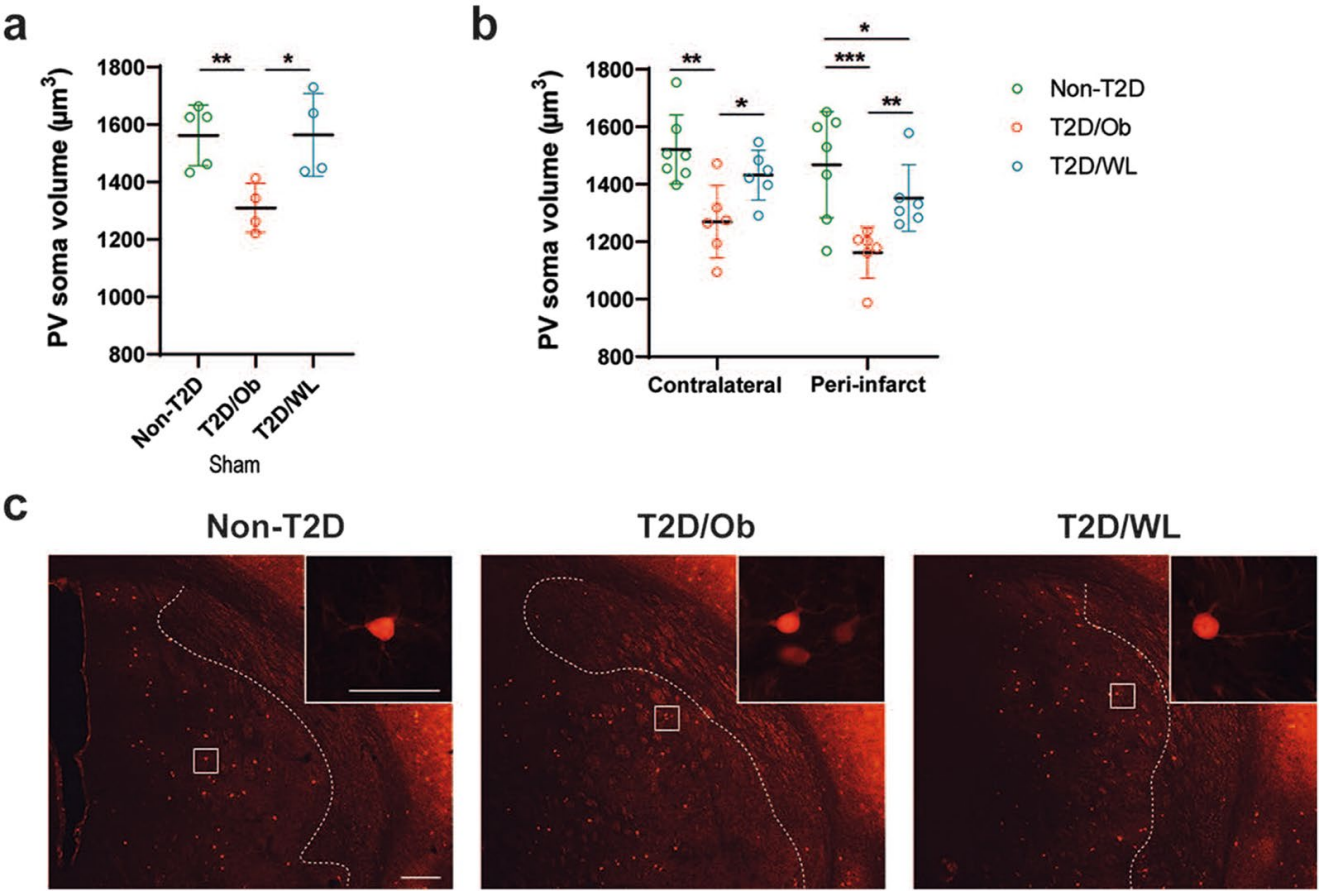
Dietary change leading to weight loss counteracts T2D-induced atrophy of PV+ interneurons. **a** Average soma volume of PV+ interneurons in striatum after sham surgery. **b** Average soma volume of PV+ interneurons in contralateral striatum and peri-infarct ipsilateral striatum after tMCAO. Data are presented as mean ± SD. Brown–Forsythe and Welch ANOVA (**a**) and Two-way ANOVA (**b**) followed by two-stage linear step-up procedure of Benjamini, Krieger and Yekutieli was used for analyses. * Denotes p < 0.05, ** denotes p < 0.01, *** denotes p < 0.001. **c** Representative images of PV+ interneurons in the ipsilateral striatum of non-T2D, T2D/Ob and T2D/WL mice after tMCAO. White dotted lines indicate the infarct region (infarct region is on right side of the dotted line; peri-infarct region is on left side of the dotted line). Scale bar for lower magnification images: 200 µm. Scale bar for higher magnification image inserts: 50 µm. Group sizes: **a** non-T2D n = 5, T2D/Ob n = 4, T2D/WL n = 4. **b** Non-T2D n = 7, T2D/Ob n = 6, T2D/WL n = 6

We previously showed that after stroke, the soma volume of PV+ interneurons is decreased in the peri-infarct region of the striatum and, while in non-T2D mice this effect is transient and the PV soma volume recovers back to normal within 6 weeks after stroke, this atrophy persists in T2D [43]. We here investigated the potential effect of long-term (4 months) dietary change leading to weight loss on atrophy of PV+ interneurons at 8 weeks after stroke. The results in Fig. 5b show a substantial atrophy of PV+ interneuron soma volume in the contralateral and ipsilateral peri-infarct striatum of T2D/Ob mice compared to the corresponding regions in non-T2D mice (p < 0.001 for contralateral striatum, and p < 0.0001 for ipsilateral peri-infarct striatum). In T2D/WL mice, this effect was almost entirely abolished (Fig. 5b, c). Interestingly, we observed the same effects in sham-operated animals (Fig. 5a). The results suggest that T2D and weight loss through dietary change, respectively, impair and restore the morphological phenotype (and likely the function) of this group of GABAergic interneurons. This effect occurs already before stroke and it persists during the whole duration of the post-stroke recovery phase.

### Pre-stroke dietary change leading to weight loss does not significantly affect stroke-induced neovascularization

**Fig. 6:**
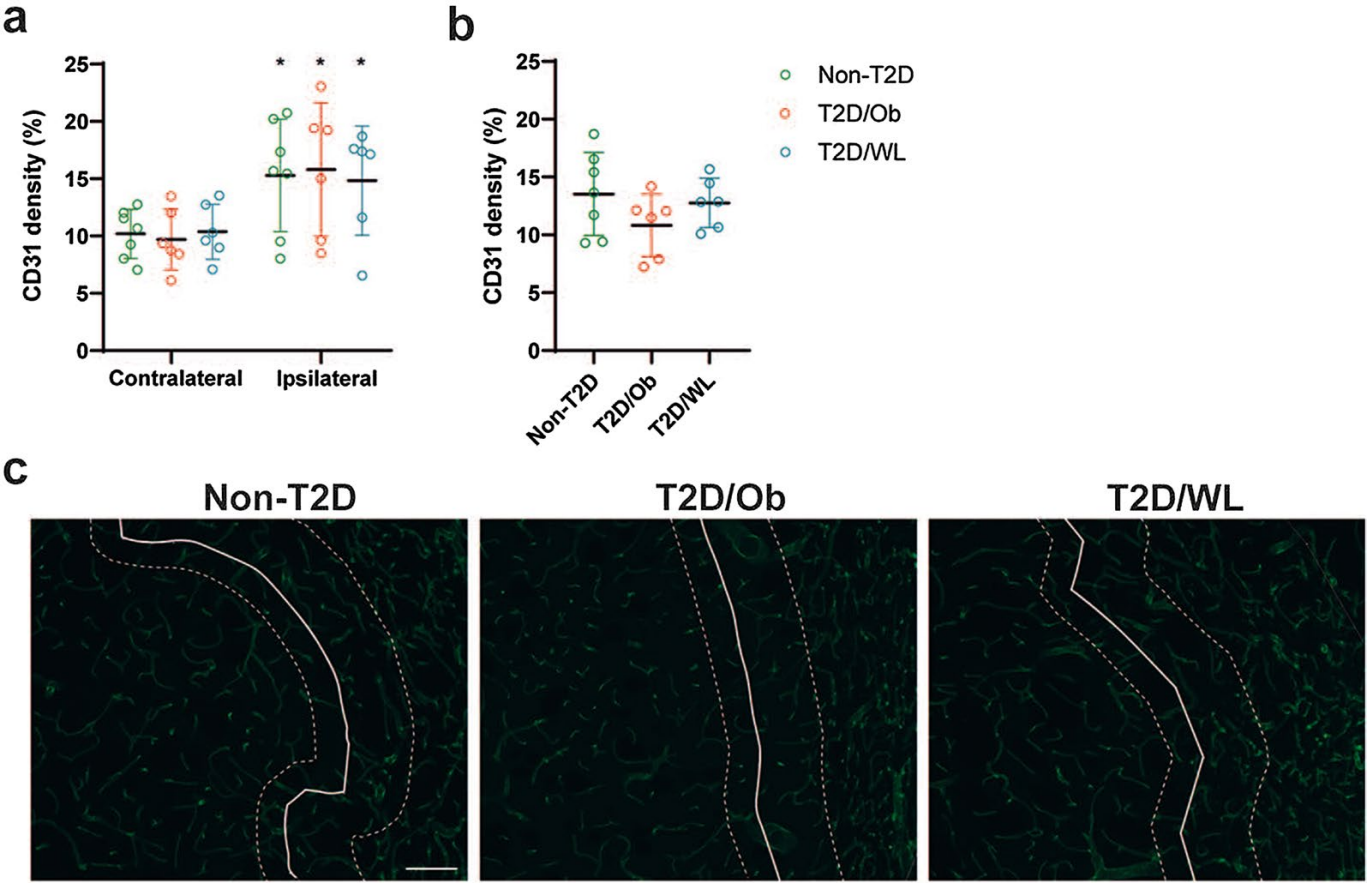
Pre-stroke T2D and dietary change leading to weight loss do not significantly alter vessel density after tMCAO. **a** CD31+ vessel density in contralateral striatum and infarct core (ipsilateral striatum) after tMCAO. **b** CD31+ vessel density in the infarct border (ipsilateral striatum) after tMCAO. Data are presented as mean ± SD. Two-way ANOVA (**a**) or Brown–Forsythe and Welch ANOVA (**b**) followed by two-stage linear step-up procedure of Benjamini, Krieger and Yekutieli was used for analyses. * Denotes p < 0.05. **c** Representative images of CD31+ vessel staining in the ipsilateral striatum of non-T2D, T2D/Ob and T2D/WL mice after tMCAO. White solid lines indicate the infarct region (infarct region is on right side of the solid line; peri-infarct region is on left side of the solid line). White dotted lines delineate the infarct border region that was analyzed in figure (**b**). Scale bar: 100 µm. Group sizes: **a**, **b** non-T2D n = 7, T2D/Ob n = 6, T2D/WL n = 6

To assess whether the improved functional recovery after long-term (4 months) weight loss was associated with changes in vascular remodeling, CD31+ vessel density was measured at 8 weeks after stroke in Study 2. A significant stroke-induced increase in vessel density was found in all groups, when comparing the contralateral striatum versus the infarct region (devoid of NeuN-positive neurons) of the ipsilateral striatum (Fig. 6a). However, no effect of T2D/Ob and T2D/WL on vessel density could be observed in the infarct region (Fig. 6a). Interestingly, a trend towards a diminished and normalized vessel density, respectively, in T2D/Ob and in T2D/WL mice, was found in the region analyzed at the border of the infarct (Fig. 6b, c). No significant differences between non-T2D, T2D/Ob and T2D/WL were found in sham mice (Additional file 1: Fig. S4).

## Discussion

The goal of this study was to determine the potential efficacy of a pre-stroke dietary change intervention leading to weight loss in T2D/obese mice to improve post-stroke recovery. A short- (2 months) and a long-term (4 months) dietary change were compared. The results show that although both strategies resulted in a similar weight loss, only the long-term (4 months) dietary change enhanced functional recovery after stroke. The effect was associated with the normalization of glucose metabolism and in particular with the reduction of insulin resistance. Stroke recovery after weight loss also occurred in association with the pre-stroke reduction of cellular atrophy of PV+ interneurons and with the decrease of stroke-induced inflammation that was enhanced by T2D in the post-stroke recovery phase.

Several studies indicate that weight loss through dietary change can effectively treat T2D [46]. Although a specific positive effect of weight loss on stroke risk has not yet been proven, weight loss-based strategies are known to prevent macrovascular disease as a whole [10, 47, 48]. Moreover, high body weight variability predicts the development of cardiovascular complications in T2D [49]. This suggests that interventions targeting the body weight could also be efficacious for stroke prevention. An important question that has not been addressed previously is whether weight loss through dietary change (or even bariatric surgery) can also improve post-stroke neurological recovery. The answer to this question is very important since T2D worsens initial stroke outcome and it is a strong predictor of post-stroke dependency on supportive care in activities of daily living [50–53]. Thus, stroke patients with T2D constitute a large group in need of effective rehabilitation therapies. Furthermore, the medical need in this area will continuously grow due to the epidemic rise of diabetes globally [2], which will increase the number of stroke patients in the near future.

We hereby show the significant improvement of functional recovery by pre-stroke weight loss induced by long-term (4 months) dietary change. We also demonstrated that despite a substantial weight loss, a short-term (2 months) dietary change is insufficient to improve recovery, likely because it does not lead to the full normalization of glucose metabolism (i.e. glycemia and IR) as a prolonged, 4 month-long dietary change does. To come to this conclusion, we compared the body weight, glycemia and IR after 2 and 4 months of dietary change leading to weight loss, both before stroke and during the entire post-stroke recovery phase. Although the final body weight after 2 and 4 months of dietary change was similar, both before and after stroke, the fasting glucose levels before stroke were different between the two experimental paradigms. Specifically, 2-months of dietary change improved pre-stroke glycemia versus T2D/Ob mice, but the glycemic levels remained above the T2D threshold of 7 mmol/L. On the contrary, 4 months of dietary change resulted in the full normalization of glycemia pre-stroke. The importance of the pre-stroke normalization of glycemia for stroke recovery is also supported by the data showing that the post-stroke glycemic levels were undistinguishable between the groups that underwent either 2- or 4-months dietary change for the whole post-stroke recovery phase.

Two months of dietary change also improved pre-stroke insulin sensitivity versus T2D/Ob mice, but similarly to glycemia, the full normalization back to the values of the non-T2D control mice was only achieved after 4 months of dietary change. Importantly (and differently from glycemia), IR after 2 months of dietary change remained also significantly higher than non-T2D mice for the whole post-stroke recovery phase, whilst in the mice after 4 months of dietary change this parameter (as the glycemia) was already fully normalized (equal to non-T2D mice) before experimental stroke.

Although correlative, these results suggest that the full normalization of pre-stroke glycemia and IR after 4-months of dietary change versus the partial normalization of these parameters after only 2-months may play a key role in improving functional recovery after stroke. Moreover, the normalization of IR after 4 months of dietary change may play a more determinant role in improving post-stroke recovery, since this parameter was only partially normalized by 2-months of dietary change also during the post-stroke recovery phase while after 4-months of dietary change insulin resistance was fully reversed. Follow-up studies should test the hypothesis of whether the administration of anti-diabetic drugs in addition to diet-induced weight loss could result in additive and/or synergistic effects on stroke recovery. Glucagon-like peptide 1 receptor agonists and dipeptidyl-peptidase 4 inhibitors are very interesting in this respect since many preclinical studies have shown their positive effects on stroke recovery [37, 39, 54]. Sodium–glucose cotransporter-2 (SGLT2) inhibitors are also very attractive drugs as they reduce the rate of adverse cardiovascular outcome and mortality in T2D patients [55]. Furthermore, they produce beneficial effects in the heart [56, 57]. However, no study has investigated the potential efficacy of SGLT2 inhibitors on stroke recovery.

In the search of potential additional metabolic factors involved in improved post-stroke recovery induced by 4 months of dietary change, we focused our studies on PAI-1 which is a key player in the regulation of fibrinolysis and several studies have shown abnormal levels of this protein in both T2D and insulin resistant states [58]. PAI-1 has been also associated with the progression of the metabolic syndrome and diabetes but also with the worsening of stroke pathophysiology [45]. As expected, we recorded a significant increase of PAI-1 in T2D/Ob mice before stroke. Interestingly, this effect was entirely abolished (back to the same levels of non-T2D mice) by 4 months of dietary change, before stroke induction. However, we could not record the same effect post-stroke, since tMCAO reduced the serum levels of PAI-1 in T2D/Ob mice to the levels of the non-T2D mice. Additional experiments will be needed to understand whether the strong pre-stroke downregulation of PAI-1 by dietary change leading to weight loss is linked to improved post-stroke functional recovery.

To identify some of the cellular mechanisms involved in improved post-stroke recovery by weight loss, we investigated T2D-induced mechanisms of neurovascular damage known to hamper functional recovery in the post-acute phase after stroke. We specifically focused on neuroinflammation, stroke-induced angiogenesis and cellular atrophy of GABAergic PV+ interneurons.

Neuroinflammation plays an important role in stroke recovery [59]. We previously found that impaired stroke recovery in T2D mice correlates with increased microglial activation during the post-stroke recovery phase [33]. Our current findings confirm this by showing increased Iba-1 immunoreactivity in the ipsilateral striatum of T2D/Ob mice as compared to non-T2D mice, at 8 weeks after stroke. Strikingly, the aggravated post-stroke microglia activation in T2D/Ob mice could be prevented entirely by 4 months of pre-stroke dietary change leading to weight loss. The beneficial effect of weight loss could also be potentially related to the reduction/normalization of HFD-induced low-grade systemic inflammation, for example the reduction of adipocyte-secreted pro-inflammatory cytokines, interleukin-1 and interleukin-6 as well as high-sensitivity CRP [60–63]. Stroke-induced reactive gliosis protects the ischemic penumbra as shown by larger infarcts in mice in which reactive gliosis was attenuated by genetic ablation of GFAP and vimentin (*GFAP*^*−/−*^*Vim*^*−/−*^) [28, 32] and *GFAP*^*−/−*^*Vim*^*−/−*^ astrocytes exhibit less efficient endothelin-3-induced blockage of gap junctional communication [28], lower levels of glutamine [64], lower glutamate transport [28] or increased vulnerability to oxidative stress [65]. Here we found no difference between the dietary groups in GFAP immunoreactivity in the post-stroke ipsilateral striatum 8 weeks after stroke. Strikingly, the GFAP immunoreactivity was increased in the contralateral striatum of T2D/Ob mice, but in the weight loss group was comparable to the sham group levels. This implies that T2D-induced post-stroke neuroinflammatory response of astrocytes in the contralateral striatum is prevented by dietary change prior to stroke. It is tempting to speculate that astrocyte reactivity in the contralesional hemisphere at 8 weeks after stroke reflects persisting neuroinflammation, which might contribute to secondary neurodegeneration. These results provide further support for the role of exacerbated neuroinflammation in the impairment of long-term functional recovery after stroke in T2D, and strongly indicate that a pre-stroke normalization of weight by a dietary change could mitigate this effect.

Vascular remodelling plays a key role in post-stroke recovery [66]. In a recent study [37], we found a significant increase in striatal vessel density in the peri-infarct region compared to the respective contralateral striatum, in both T2D/Ob and non-T2D mice at 8 weeks post-stroke, indicating a post-stroke angiogenic effect. However, this effect was smaller in the T2D/Ob group. In the current study, we only observed a trend towards a decreased angiogenic response in the T2D/Ob group and interestingly this trend was reversed in the T2D/WL group. Although speculative, these results suggest a potential role of a 4-month dietary change leading to weight loss before stroke in restoring the vascular dysfunction caused by T2D in the infarct region. If so, this effect could be potentially associated with the restoration of the long-term functional recovery after stroke. However, additional studies are needed to prove this hypothesis.

PV+ interneurons in striatum play a key role in facilitating movement by modulating the activity of striatal medium spiny neurons [67]. Impaired functioning of PV+ neurons after stroke may therefore contribute to stroke pathology, by causing a disturbed excitation-inhibition balance in brain circuits [68]. We have observed in several studies that T2D/Ob causes atrophy of PV+ interneurons in the ipsilateral striatum after stroke [33, 37, 69]. The current study confirms these findings. Moreover, T2D/Ob-induced atrophy of PV+ interneurons was also found in the contralateral striatum, as well as in striatum of sham T2D/Ob mice. As such, it appears that T2D causes atrophy of striatal PV+ interneurons and that this effect persists after stroke. Although speculative, we hypothesize that T2D hampers post-stroke functional recovery by, among other effects, decreasing the soma size of PV+ interneurons both pre- and post-stroke. Indeed, reduced neuronal soma size has been related with diminished neuronal activity and connectivity [70–73] and decreased neuronal soma volume has also been linked with different diseases, including Huntington’s disease, Down syndrome, Rett syndrome and schizophrenia [71, 73–76]. Accordingly, the beneficial effects of pre-stroke weight loss via dietary change on post-stroke recovery might rely on rescuing PV+ interneurons from T2D-induced atrophy, since weight loss was found to normalize PV+ interneuron soma size in both sham and stroke mice. We are aware that measuring atrophy of PV+ interneurons does not provide direct insights into their functioning and/or activity. Therefore, whether T2D and weight loss induced by dietary change can respectively impair and improve functional recovery after stroke through changes in the soma volume of PV+ interneurons remain to be addressed by functional studies.

## Conclusions

The number of diabetic people suffering from stroke is continuously growing and today, more than ever, stroke neurologists are challenged to deeply understand how to treat stroke patients suffering from diabetes. The idea of “treating diabetes to prevent stroke” is very tangible in this respect and supported by several studies [77]. This study extends this concept to promote future clinical trials aimed at determining if treating diabetic people with dietary change leading to weight loss will improve their neurological recovery after stroke. If so, this will facilitate their rehabilitation process and aid secondary prevention. Our results strongly support this type of strategy and specifically suggest that a long-term weight loss able to normalize glucose metabolism and in particular IR can have profound effects in the brain by reducing neuroinflammation, enhancing neuroplasticity and leading to improved functional recovery after stroke.

## Supplementary Information

**Additional file 1: Figure S1.** T2D and dietary change leading to weight loss do not significantly affect stroke volume. **Figure S2.** Comparison of the effects of short-term vs. long-term dietary change on weight and glucose metabolism. **Figure S3.** Dietary change leading to weight loss reverses T2D-induced increased PAI-1 plasma levels. **Figure S4.** Pre-stroke T2D and weight loss do not significantly alter vessel density in sham mice. Additional material: immunohistochemistry; analysis of PV+ interneuron cell body volume; analysis of vessel density.

## Data Availability

The data that support the findings of this study are available from the corresponding authors upon reasonable request.

## Abbreviations

AUC: Area under the curve
BW: Body weight
CD31: Cluster of differentiation 31/platelet endothelial cell adhesion molecule
CD68: Cluster of differentiation 68
ECA: External carotid artery
GABA: Gamma aminobutyric acid
HFD: High-fat diet
Iba-1: Ionized calcium-binding adapter molecule 1
ICA: Internal carotid artery
ITT: Insulin tolerance test
IR: Insulin resistance
MCA: Middle cerebral artery
NeuN: Neuronal nuclei
PAI-1: Plasminogen activator inhibitor 1
PBS: Phosphate-buffered saline
SD: Standard laboratory diet
SGLT2: Sodium-glucose cotransporter 2
T2D: Type 2 diabetes
tMCAO: Transient middle cerebral artery occlusion
