# Supplementary material for: Diet-induced weight loss in obese/diabetic mice normalizes glucose metabolism and promotes functional recovery after stroke
Source: Cardiovasc Diabetol. 2021 Dec 22;20:240. doi: 10.1186/s12933-021-01426-z (PMC8697500; doi:10.1186/s12933-021-01426-z)
Supplement: Supplementary file 1 — Additional file 1: Figure S1. T2D and dietary change leading to weight loss do not significantly affect stroke volume. Figure S2. Comparison of the effects of short-term vs. long-term dietary change on weight and glucose metabolism. Figure S3. Dietary change leading to weight loss reverses T2D-induced increased PAI-1 plasma levels. Figure S4. Pre-stroke T2D and weight loss do not significantly alter vessel density in sham mice. Additional material: immunohistochemistry; analysis of PV+ interneuron cell body volume; analysis of vessel density. [file 12933_2021_1426_MOESM1_ESM.docx]

**Diet-induced weight loss in obese/diabetic mice normalizes glucose metabolism and promotes functional recovery after stroke**

Dimitra Karampatsi^1^, Alexander Zabala^1^, Ulrika Wilhelmsson^2^, , Doortje Dekens^1^, Ellen Vercalsteren^1^, Martin Larsson^1^, Thomas Nyström^1^, Milos Pekny^2^, Cesare Patrone**^**^1^, Vladimer Darsalia**^**^1^

^1^NeuroCardioMetabol Group, Department of Clinical Science and Education, Södersjukhuset, Internal Medicine, Karolinska Institutet, 118 83 Stockholm, Sweden

^2^Laboratory of Astrocyte Biology and CNS Regeneration, Center for Brain Repair and Rehabilitation, Department of Clinical Neuroscience and Rehabilitation, Institute of Neuroscience and Physiology, Sahlgrenska Academy at the University of Gothenburg, Sweden

Dimitra Karampatsi (M.Sc.) E-mail [dimitra.karampatsi@ki.se](mailto:dimitra.karampatsi@ki.se)

Alexander Zabala (M.D.) E-mail [alexander.zabala@ki.se](mailto:alexander.zabala@ki.se)

Ulrika Wilhelmsson (Ph.D.) [ulrika.wilhelmsson@neuro.gu.se](mailto:ulrika.wilhelmsson@neuro.gu.se)

Doortje Dekens (Ph.D.) E-mail [doortje52@hotmail.com](mailto:doortje52@hotmail.com)

Ellen Vercalsteren (Ph.D.) E-mail ellen.vercalsteren@ki.se

Martin Larsson (M.D., Ph.D.) E mail martin.k.larsson@regionstockholm.se

Thomas Nyström (M.D., Ph.D.) E mail [thomas.nystrom@ki.se](mailto:thomas.nystrom@ki.se)

Milos Pekny (M.D., Ph.D.) [milos.pekny@neuro.gu.se](mailto:milos.pekny@neuro.gu.se)

Cesare Patrone (Ph.D.) E-mail [cesare.patrone@ki.se](mailto:cesare.patrone@ki.se)

Vladimer Darsalia (Ph.D.) E-mail [vladimer.darsalia@ki.se](mailto:vladimer.darsalia@ki.se)

**CORRESPONDING AUTHORS:**

^Cesare Patrone (Ph.D.), Karolinska Institutet, Department of Clinical Science and Education, Södersjukhuset, 118 83 Stockholm, Sweden. Phone: +46 (8) 6165084. Fax: +46 (8) 6162933. ORCID: [0000-0003-0470-4606](javascript:popup_orcidDetail('https://orcid.org','0000-0003-0470-4606');)

^Vladimer Darsalia (Ph.D.), Karolinska Institutet, Department of Clinical Science and Education, Södersjukhuset, 118 83 Stockholm, Sweden. Phone: +46 (8) 6165084. Fax: +46 (8) 6162933. ORCID: 0000-0002-6693-934X

**Additional Figures**

**
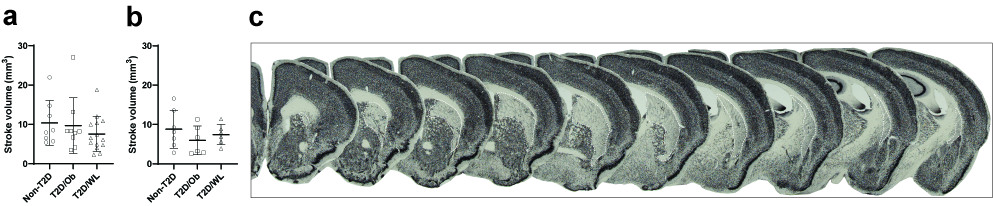
**

**Fig. S1** T2D and dietary change leading to weight loss do not significantly affect stroke volume.

a: Ischemic volume in Study 1. b: Ischemic volume in Study 2. c: Representative images of NeuN staining. The white dotted lines on images indicate stroke area. Brown-Forsythe and Welch ANOVA followed by two-stage linear step-up procedure of Benjamini, Krieger and Yekutieli was used to analyse ischemic volume. Data are presented as mean ± SD. Group sizes: a: Non-T2D n=8, T2D/Ob n=9, T2D/WL n=13. b: Non-T2D n=7, T2D/Ob n=6, T2D/WL n=6.


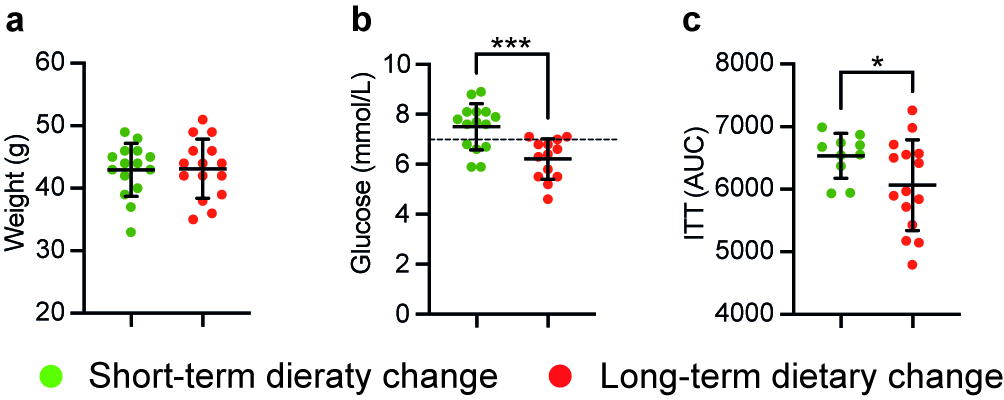


**Fig. S2** Comparison of the effects of short-term vs. long-term dietary change on weight and glucose metabolism.

a: weight. b: fasting glucose. c: insulin sensitivity. Welch’s t-test. Group sizes: Short-term dietary change n=15, Long-term dietary change n=15.

**
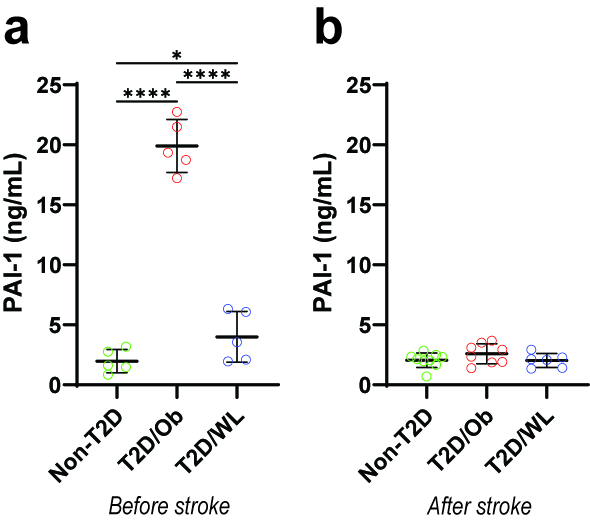
**

**Fig. S3** Dietary change leading to weight loss reverses T2D-induced increased PAI-1 plasma levels.

Plasma levels of PAI-1 before (a) and after (b) tMCAO. Data are presented as mean ± SD. Brown-Forsythe and Welch ANOVA followed by two-stage linear step-up procedure of Benjamini, Krieger and Yekutieli was used to analyse differences. * denotes p < 0.05, **** denotes p < 0.0001. Group sizes: a: Non-T2D n=5, T2D/Ob n=5, T2D/WL n=5. b: Non-T2D n=9, T2D/Ob n=8, T2D/WL n=6.


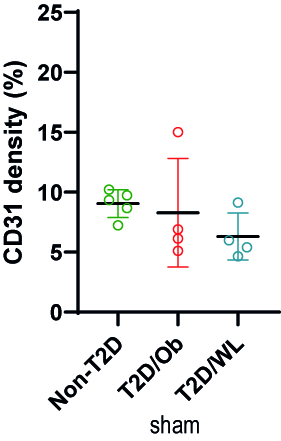


**Fig. S4** Pre-stroke T2D and weight loss do not significantly alter vessel density in sham mice.

CD31+ vessel density in striatum of sham mice. Data are presented as mean ± SD. Brown-Forsythe and Welch ANOVA followed by two-stage linear step-up procedure of Benjamini, Krieger and Yekutieli was used. Group sizes: Non-T2D n=5, T2D/Ob n=4, T2D/WL n=4.

**Additional Material**

*Immunohistochemistry*

The following primary antibodies were used; mouse anti-NeuN (1:500 dilution, #MAB377, Millipore; RRID:AB_2298772), a neuronal marker; goat anti-Iba-1 (1:1000 dilution, #ab5076, Abcam; RRID:AB_2224402), a marker for microglia; rabbit anti-CD68 (1:2000 dilution, #ab125212, Abcam; RRID:AB_10975465), a marker for phagocytic microglia and macrophages; mouse anti GFAP (1:500 dilution, #173011, Synaptic Systems; RRID:AB_2232308), a marker for astrocytes; rabbit anti-parvalbumin (PV) (1:1500 dilution, #ab11427, Abcam; RRID:AB_298032), a marker of parvalbumin-expressing interneurons; goat anti-CD31 (1:200 dilution, #AF3628, R&D Systems; RRID:AB_2161028), a marker for endothelial cells. The following primary antibodies were used; mouse anti-NeuN (1:500 dilution, #MAB377, Millipore; RRID:AB_2298772), a neuronal marker; goat anti-Iba-1 (1:1000 dilution, #ab5076, Abcam; RRID:AB_2224402), a marker for microglia; rabbit anti-CD68 (1:2000 dilution, #ab125212, Abcam; RRID:AB_10975465), a marker for phagocytic microglia and macrophages; mouse anti GFAP (1:500 dilution, #173011, Synaptic Systems; RRID:AB_2232308), a marker for astrocytes; rabbit anti-parvalbumin (PV) (1:1500 dilution, #ab11427, Abcam; RRID:AB_298032), a marker of parvalbumin-expressing interneurons; goat anti-CD31 (1:200 dilution, #AF3628, R&D Systems; RRID:AB_2161028), a marker for endothelial cells.

The following secondary antibodies were used: biotinylated horse anti-mouse (1:200 dilution, #BA-2000, Vector Laboratories; RRID:AB_2313581); biotinylated horse anti-goat (1:200 dilution, #BA-9500, Vector Laboratories; RRID:AB_2336123); biotinylated horse anti-rabbit (1:200 dilution, #BA-1100, Vector Laboratories; RRID:AB_2336201); horse anti-rabbit DyLight 594 (1:200 dilution, #DI-1094, Vector Laboratories; RRID:AB_2336414); horse anti-mouse DyLight 488 (1:200 dilution, #DI-2488, Vector Laboratories; RRID:AB_2307439), donkey anti mouse Alexa fluor 555 (1:1000 dilution, # A31570, Invitrogen; RRID: AB_2536180); donkey anti-goat Alexa fluor 488 (1:200 dilution, #A11055, Invitrogen; RRID:AB_2534102).

*Analysis of PV+ interneuron cell body volume*

The Optical Fractionator Workflow was started, and the region of interest was delineated (whole striatum for sham mice and contralateral striatum, and peri-infarct striatum for ipsilateral striatum (based on NeuN staining)) at 4x magnification. The microscope was then switched to 40x magnification, to enable measurement of PV+ interneuron cell body volume with the Nucleator tool (isotropic uniform random sections, 5 arms/rays for each nucleator measurement). PV+ interneurons were analysed at software-determined (experimenter-independent) random positions within the region of interest. Three sections were measured per mouse, and at least 50 interneurons were analysed per mouse. The average cell body volume was automatically calculated by the Stereo Investigator software (using the manually entered average mounted section thickness of 30 µm).

*Analysis of vessel density*

Blood vessel (CD31+) density was analysed using the area fraction measurement tool of ImageJ software (NIH, USA). The density was expressed as the percentage of the CD31+ area. In the ipsilateral striatum, CD31 density was analysed in two different regions of interest. Firstly, CD31 density was measured within the infarct region (the region devoid of NeuN-positive neurons). Secondly, CD31 density was measured at the border of the infarct. For this, we analysed a region of approximately 200 µm in width that overlapped the infarct and peri-infarct region (2/3 part infarct, 1/3 part peri-infarct).
